# Bias against AI art can enhance perceptions of human creativity

**DOI:** 10.1038/s41598-023-45202-3

**Published:** 2023-11-03

**Authors:** C. Blaine Horton Jr, Michael W. White, Sheena S. Iyengar

**Affiliations:** Columbia Business School, New York, USA

**Keywords:** Psychology, Human behaviour

## Abstract

The contemporary art world is conservatively estimated to be a $65 billion USD market that employs millions of human artists, sellers, and collectors globally. Recent attention paid to AI-made art in prestigious galleries, museums, and popular media has provoked debate around how these statistics will change. Unanswered questions fuel growing anxieties. Are AI-made and human-made art evaluated in the same ways? How will growing exposure to AI-made art impact evaluations of human creativity? Our research uses a psychological lens to explore these questions in the realm of visual art. We find that people devalue art labeled as AI-made across a variety of dimensions, even when they report it is indistinguishable from human-made art, and even when they believe it was produced collaboratively with a human. We also find that comparing images labeled as human-made to images labeled as AI-made increases perceptions of human creativity, an effect that can be leveraged to increase the value of human effort. Results are robust across six experiments (*N* = 2965) using a range of human-made and AI-made stimuli and incorporating representative samples of the US population. Finally, we highlight conditions that strengthen effects as well as dimensions where AI-devaluation effects are more pronounced.

## Will AI art devalue human creativity?

The contemporary art world is conservatively estimated to be a $65 billion USD market that employs millions of human artists, sellers, and collectors across the world^1^. Yet recent attention paid to art made by artificial intelligence (AI) in prestigious galleries^2,3^, museums^4^, and popular media^5^ has provoked heated debate around how these statistics will change in the future^6,7^. Anxiety around the changing value of human art is fueled by unanswered questions: Will art attributed to AI be evaluated in the same way as art attributed to humans? Should art markets even treat AI-made art as “art”? Does growing exposure to AI-made art impact evaluations of solitary human creativity or, as is also happening, evaluations of human artists using AI?

Historical examples from other industries provide ample evidence that, on average, automation decreases the value of human goods and labor^8^. But there is also reason to believe that the development of AI technologies capable of automating creativity (e.g., producing visual art largely indistinguishable from human art) should not similarly impact perceptions of human art and creativity. As the famous American painter James Whistler once said, "An artist is not paid for his labor but for his vision"^9^. Past research supports Whistler’s point. Whether made by experts or lay audiences, evaluations of art often depend upon both aesthetic and social dimensions that can be disentangled from the more tangible costs of production and labor^10,11^. For example, an artist’s use of color and emotion, the complexity of their subject matter, and the artist’s brand all impact perceptions of value and creativity assigned to a given piece of art^12,13^. Subjective factors like these make it difficult to predict whether growing exposure to AI-made art will negatively impact the value of human creativity in artistic domains. Put another way, will the value of human art (monetary and aesthetic) increase or decrease when evaluated next to works (of comparable artistic style and quality) thought to be produced by AI programs? The growing importance of questions like this are reflected in headlines that detail how some human artists have recently begun to take legal action against AI companies upon discovering AI programs are being used to emulate their unique artistic styles with startling accuracy^14^. Furthermore, examples like these fuel larger concerns that the value of human labor may be changing. Is it true that, as some have suggested, creative jobs are “the last bastion of humanity”^15^, or will AI decrease the value of human labor as has happened in so many other industrial revolutions of the past^8^? As a more comprehensive review on this topic suggests, scientific inquiries focusing specifically on the intersections of technology and creativity are needed if we are to understand the impact generative AI is beginning to have on the world^16^.

The current research adopts a psychological perspective to begin answering these questions. Specifically, we examine how artistic source attributions (e.g., human-made or AI-made) influence evaluative judgments of lay audiences across six experiments (total *N* = 2965). To adopt a comprehensive view, we assess an array of different dimensions that include estimates of monetary value and labor as well as more *artistic* evaluations, such as perceptions of skill and creativity. Our focus on art builds upon past work suggesting humans often exhibit costly aversions to decision-making algorithms, preferring instead to rely on humans for a variety of goods and services, even when algorithms performs better than or on par with human agents^17–19^. Art presents a unique domain of exploration in this field, in part because recent research suggests humanizing autonomous technologies can help mitigate aversion to algorithms^20–23^. This finding highlights a philosophical quandary. What is more humanizing than the ability to produce art? Indeed, many famed artists and scientists across the ages have proclaimed art to be a fundamentally human pursuit (e.g., James Joyce once said “art is the human disposition”^24^ and the esteemed scientist Brian Greene similarly remarked “art [is what] makes us human”^25^). If true, these philosophical notions suggest that artistic works attributed to AI should not be subject to the algorithmic aversion observed in past work because the ability to produce art may, in and of itself, be humanizing. On the other hand, it is also possible that art attributed to AI will instead begin to change the ways we perceive and evaluate *human* creativity going forward. While the breadth of these speculations extends well beyond the confines of any single scientific examination, our research presents three key findings that shed new light on this topic.

First, participants in our experiments consistently devalued art labeled as AI-made relative to art labeled as human-made. This was true even when the art in question was largely indistinguishable from the art of famed human artists and when we held the art itself constant (i.e., labeling the exact same piece as either “human-made” or “AI-made”). These effects were also evidenced regardless of participants’ overall feelings towards AI or their background experiences (e.g., education or profession) focused on art or technology. These points are important, because many of the effects observed in past work on algorithmic aversion can be explained either by straightforward confounds like simple differences in content of stimuli or by unmeasured, individual differences like participant dispositions towards new technologies^26^. Even accounting for these, our first key finding continues to echo many historical examples of automation in other industries, with devaluation effects being particularly pronounced on evaluations of skill and monetary value. Qualifying this, however, is the fact that effects were noticeably less pronounced on more artistic, aesthetic dimensions (e.g., evaluations of complexity or emotional intensity) and weakened substantively when participants were not asked to directly compare human and AI-made efforts.

Our second key finding was that art labeled as human-made was seen as *more* creative as a function of exposure to art labeled as AI-made. That is, the same piece of art gained creative value when it was labeled as human-made and compared to other works of art labeled as AI-made relative to when it was labeled as human-made and compared to other works of art labeled as human-made. This finding is both surprising and important, because contrary to predictions expressed in popular media outlets, it indicates there is potential for human artists to benefit from comparisons made between their work and the work of AI artists. It suggests novel avenues of thought, among them the idea that AI programs may also represent tools that can be used to highlight or accentuate the creative capacity of humans going forward.

Our third and final key finding is that although art described as collaboratively made (i.e., art created by human artists working with AI programs) was, on average, perceived to be less valuable than work described as human-made and perceived to be more valuable than work described as AI-made, perceptions of the human artist as the primary creative agent depended largely upon whether the collaboration was being compared to human or AI references. Put another way, the evaluative bias against AI-labeled artwork persisted even in circumstances where the AI functioned only as a human aid, but perceptions of the human artist's contribution within the collaboration were higher when evaluators were first anchored on the efforts of AI art produced without the help of humans. This finding is important because it suggests human artists working with AI can benefit from drawing comparisons between their collaborative output and the output of AI programs working alone.

## Experiment 1

### Results and discussion

**Figure 1:**
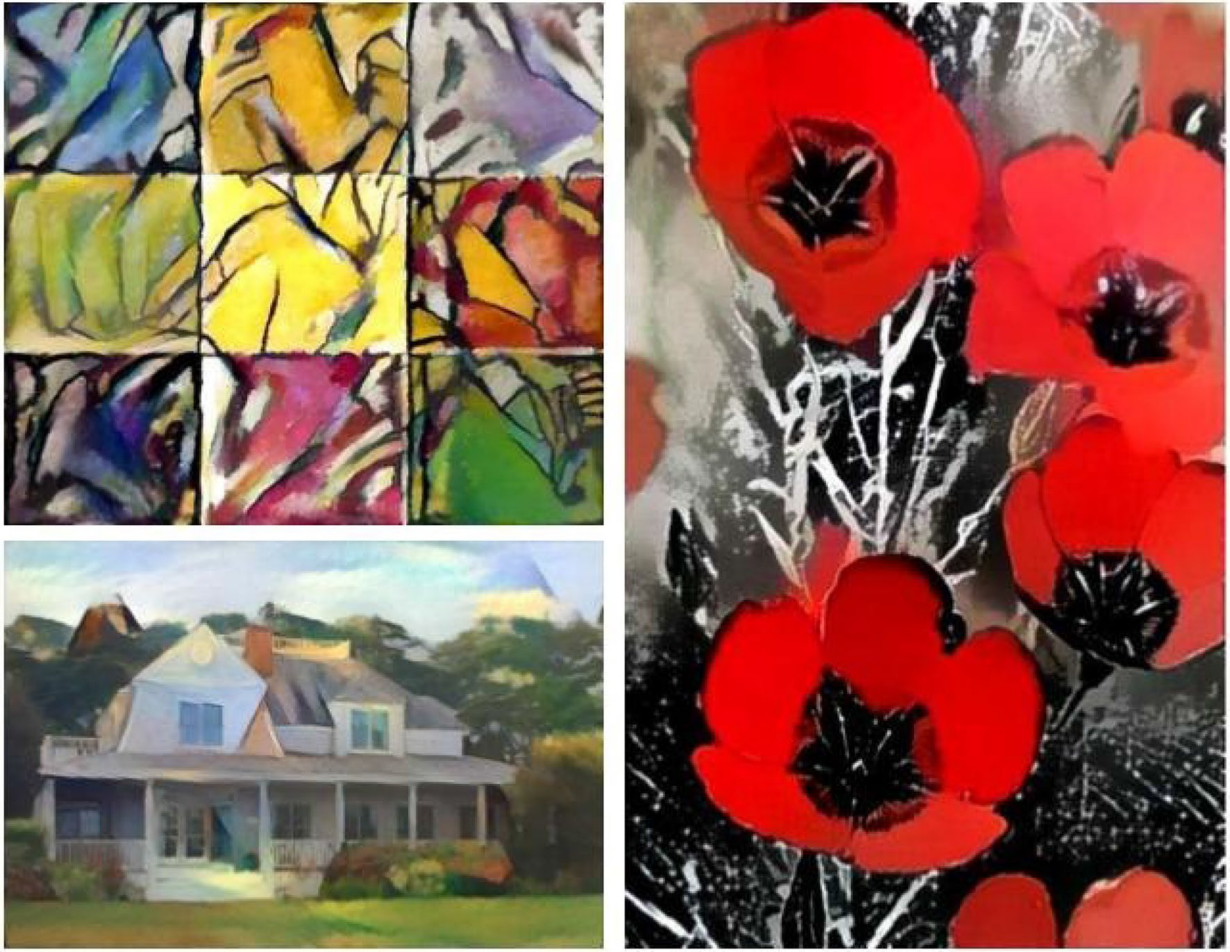
Examples of stimuli used in Experiment 1. *Note* These images represent a sample from the larger pool of 28 images used in our first study.

In Experiment 1, participants (*n* = 119) were presented with 28 different images (*observations* = 3332) that were pretested to capture a range of different artistic styles (see Fig. 1). Each image was randomly labeled such that every participant evaluated 14 images labeled as AI-made and 14 images labeled as human-made. These were evaluated on a battery of artistic dimensions that included how bright, colorful, complex, emotional, skillful, inspiring, expensive, and likable participants found each image to be (α = 0.97).

Table 1 displays our results. Overall, within-subjects comparisons revealed images labeled as AI-made were evaluated less favorably, with an aggregate effect of *t*(118) = 5.52, *p* < 0.001, *m*_*diff*_ = − 0.18, 95% CI [− 0.12, − 0.25], *d* = 0.51. The direction and significance of this effect remained unchanged when considering each dimension separately (Table 1) and when using multilevel regression models to control for variance attributable to specific image-styles or individual preferences (i.e., models controlling for image effects and nesting within participant). This effect was most pronounced on evaluations of expensiveness (*m*_*diff*_ = − 0.45, *d* = 0.57) and skill (*m*_*diff*_ = − 0.30, *d* = 0.48)—and occurred despite the majority of participants (> 70%) reporting they would not have been able to differentiate between images without the labels provided. That is, even though most participants reported art labeled as AI-made was largely indistinguishable from art labeled as human-made, they still evaluated art labeled as AI-made less favorably. These results suggest a general bias against AI-made art.

**Table 1:** Experiment 1 within-subject evaluation differences between human and AI-labeled images.

Dimension	Mean difference	Effect size (Cohen’s d)
Expensive	− 0.45***	0.57
Skillful	− 0.30***	0.48
Complex	− 0.18***	0.36
Colorful	− 0.12**	0.30
Liking	− 0.12**	0.25
Emotional	− 0.12***	0.27
Bright	− 0.08*	0.21
Inspiring	− 0.09*	0.22
Overall (α = 0.95)	− 0.18***	0.51

*P*-values reflect paired *t*-tests comparing average ratings by participants on images labeled as Human-made vs. images labeled AI-made.

*p < 0.05, **p < 0.01, ***p < 0.001.

## Experiment 2

### Results and discussion

In Experiment 2, participants (*n* = 415) evaluated the same 28 images (*observations* = 11,620) on the same evaluative dimensions used in Experiment 1 alongside an additional “willingness to pay” measure (α = 0.88). Two other key changes were made. First, we expanded our research focus by asking participants to report whether they believed each image qualified as “art” or not (1 = *no*, 2 = *maybe*, 3 = *yes*). Second, we used a between-subjects design to provide a more conservative estimate of the bias observed in Experiment 1. Participants were randomly assigned to one of three conditions where either (a) all images were unlabeled so as not to prime any thoughts about the differences between human and non-human art, (b) all images were labeled as AI-made, or (c) participants were told in advance that some images were made by humans and others by AI but not which ones (i.e., a ‘mystery’ condition that drew attention to ambiguous source information without using any labels). The first condition was used as a control condition, under the presumption that the majority of our participants would assume the unlabeled images were human-made. This presumption was based upon a pre-test of these images (conducted before our first experiment), where participants indicated that when unlabeled, a majority of the images looked human-made (see Fig. S1 in our Supplemental Information). Nevertheless, the lack of an explicit manipulation check in this condition is one limitation of this design, something addressed in later studies where more explicit labels are used.

Kruskal–Wallis tests indicated overall differences between conditions on aesthetic dimensions (*χ*^2^ [2] = 6.2, *p* = 0.04) as well as differences in how likely participants were to classify images as art (*χ*^2^ [2] = 63.19, *p* < 0.001). Means and standard errors for all dimensions and conditions are presented in Table S1 in our Supplementary Information and Dunn’s test pairwise comparisons assessing all dimensions and conditions are presented on Table S2 in our Supplementary Information. Summarizing some of our key findings here, the overall differences in evaluations were largely explained by more specific differences in participant's evaluations of how expensive (*χ*^2^ [2] = 57.19, *p* < 0.001) and skillful (*χ*^2^ [2] = 38.73, *p* < 0.001) images were perceived to be. Specifically, images labeled as AI-made were rated as being significantly less expensive (*Z* = − 7.06, *p* < 0.001) and skillful (*Z* = − 5.59, *p* < 0.001) relative to unlabeled images, and also less expensive relative to mystery images (Z = − 5.95, *p* < 0.001). Moreover, participants were less likely to say that AI-labeled images qualified as art compared to both unlabeled images (*Z* = − 7.11, *p* < 0.001) and mystery images (*Z* = − 6.70, *p* < 0.001). One caveat, though, is that many of the participants in the AI-labels condition (87%) still considered the vast majority of images to qualify as art. Similar to images labeled as AI-made, participants rated mystery images (i.e., images we said could be either human or AI-made) as significantly less skillful (*Z* = − 5.16, *p* < 0.001) and marginally less expensive (*Z* = − 1.17, *p* = 0.07), but also found them more inspiring (*Z* = 4.25, *p* = 0.07) in contrast to unlabeled images and were no less likely to consider the images to be art (*Z* = − 0.47, *p* *≈* 1). We propose these varied outcomes can be attributed to participants accurately assuming some images in the mystery condition were human-made and others AI-made. Indeed, the wider, more bimodal distributions observed in this condition serve as a manipulation check suggesting comparisons between art thought to be human and AI-made does not lead to the categorical devaluation of *all* art presented (when the source is ambiguous), but is instead encouraged by presenting clear targets in the form of labels.

In sum, these findings help to clarify results from our first experiment, confirming that the bias against AI-made art is particularly pronounced on dimensions of artistic skill and monetary value, with participants reporting that images explicitly labeled as AI-made are less likely to qualify as art. Also noteworthy, is that many within-subject differences on other evaluative dimensions that were observed in our first experiment (e.g., ratings of complexity or colorfulness) were not significant in this more conservative, between-subjects design. This may suggest the strong bias against art labeled as AI-made observed in our first experiment was less pronounced in this experiment precisely because we did not force participants to make back-to-back comparisons of images explicitly labeled as both human and AI-made.

## Experiment 3

### Results and discussion

In Experiment 3, we examine how evaluations of human creativity are impacted by immediate comparisons made between art labeled as human and AI-made. Specifically, we randomize the order of image labels to assess how human artists are evaluated *after* exposure to AI-made art. All participants (*n* = 405) evaluated two images selected from the stimuli used in our previous experiments. These two images were selected because they had comparable styles and were rated similarly across artistic dimensions (see Fig. S2 in our Supplementary Information). Participants were randomly assigned to either a control condition (a) where both paintings were explicitly labeled as human-made, or one of three experimental conditions where (b) both paintings were labeled as AI-made, (c) the first painting was labeled human-made and the second painting as AI-made, or (d) the first painting was labeled as AI-made and the second painting as human-made. Holding the images constant in this way allowed us to determine the extent to which a single piece of art gains or loses value as a function of comparing art labeled as human or AI-made. In addition to evaluating aesthetic dimensions, participants estimated the monetary value of each painting, as well as the skill, talent, and execution shown by the labeled artist (human or AI). We also told participants these images were all physical paintings currently for sale at a private gallery and included fake information provided by that gallery (e.g., “*Gallery ID: #A2461 Untitled, 2019 Oil on canvas 24 in* × *36 in*”). This was done for two reasons. First, it helped us to verify that results observed in our previous experiments could not be explained by assumptions that AI-produced paintings were simply digital images. Second, it ensured our results are generalizable to real-world markets where physical (and not just digital) pieces of art are bought and sold.

Aligned Rank Transformed Contrast (ART-C) tests were used to analyze data because they have been shown to be a particularly well suited parametric test for comparing groups and have demonstrated more power than *t* tests, Mann–Whitney, Wilcoxon, and the standard ART ANOVAs without inflating Type I error rates^27^. Our analysis revealed main effects of label, such that both paintings were evaluated as being worth less money when labeled as AI-made (*t*[403] = − 7.04, *p* < 0.001; *t*[403] = − 5.75, *p* < 0.001), with the presumed AI artist also being seen as less capable on dimensions of skill, talent, and execution (see Tables S3 and S4 in our Supplementary Information). As with Experiment 2, we did not observe an effect of labels on aesthetic dimensions such as how colorful or complex the images were (see Table S3 in our Supplementary Information). Again, we interpret this as suggesting the more pronounced devaluation effects in Experiment 1 which encompassed aesthetic dimensions were driven in part by forcing participants to make back-to-back comparisons of images labeled as both human and AI-made (i.e., using a within vs. between-subjects design). Finally, rather than detracting from evaluations of human creativity, comparisons between art labeled as human and art labeled as AI-made positively impacted evaluations of human effort (see Table S4 in our Supplementary Information). Specifically, the second painting was rated higher on aesthetic dimensions when it was labeled as human-made and followed a painting labeled as AI-made (*t*[401] = 3.30, *p* = 0.001). That is, the exact same painting was evaluated more favorably when labeled as human-made and compared against art labeled as AI-made than when it was labeled as human-made and compared against another painting labeled as human-made.

## Experiment 4

### Results and discussion

Experiment 4 was preregistered and conducted to conceptually replicate our findings from Experiment 3. Specifically, are evaluations of human creativity positively impacted by exposure to artwork attributed to AI? Participants (*n* = 789) were shown the same two images used in Experiment 3 but in place of aesthetic dimensions they were asked to evaluate creativity directly. Specifically, participants rated each painting on how creative, novel, likable, and appropriate to be sold in a gallery it was. These measures were selected based upon past research^28,29^ to reflect the “standard” definition of creativity in psychology which posits that ideas and objects are creative when they are perceived to be creative, novel, and appropriate to some goal (e.g., appropriate to be sold in galleries or to be enjoyed by audiences^29,30^). These items were averaged together to construct an overall measure of creativity (Image 1 α = 0.78, Image 2 α = 0.82). Like Experiment 3, participants were randomly assigned to either a control condition (a) where both paintings were explicitly labeled as human-made, or one of two experimental conditions where (b) the first painting was labeled human-made and the second painting as AI-made, or (c) the first painting was labeled as AI-made and the second painting as human-made. Participants were also asked to estimate the monetary value and production-time of each painting (i.e., how much time in hours they thought it took to produce each painting). Finally, for use as control variables in supplementary models, participants indicated their artistic and technological backgrounds on items like, “I used to (or currently) work in a job that primarily deals with the visual arts (e.g. designer, gallery manager, art dealer).”

**Figure 2:**
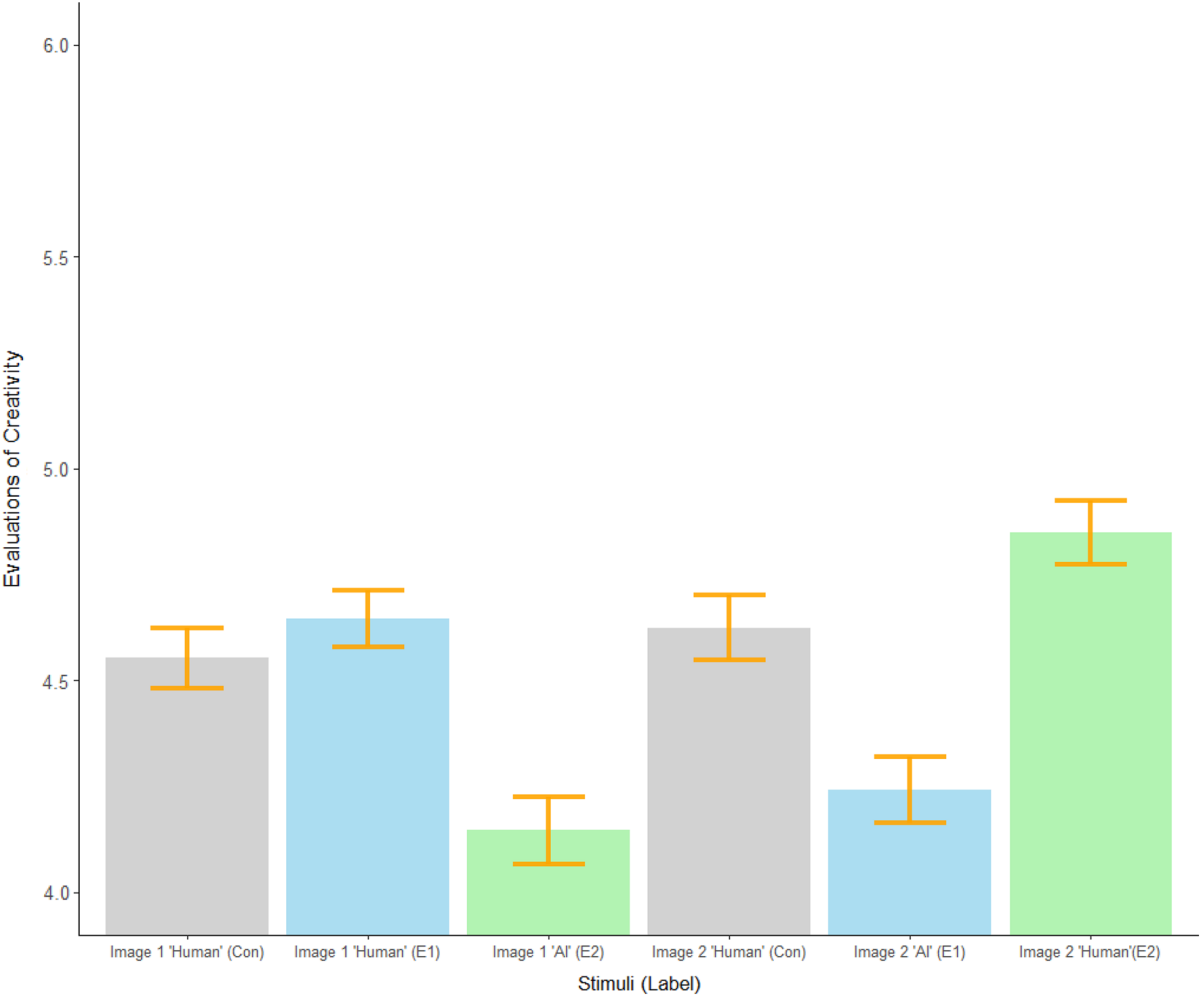
Experiment 4 evaluations of creativity by order and condition. *Note* Colors correspond to conditions. Grey is used for the control condition ("Con") which only contained images labeled as human-made. Blue is used for the first experimental condition ("E1") which contained an image labeled as human-made first and an image labeled as AI-made second. Green is used for the second experimental condition ("E2") which contained an image labeled as AI-made first and an image labeled as human-made second. The error bars represent the standard errors.

Overall, both paintings in Experiment 4 were rated as less creative, worth less money, and estimated to have taken less time to produce when labeled as AI-made (see Table 2 below and Table S5 in our Supplementary Information Material). Consistent with the positive effect on aesthetic dimensions observed in Experiment 3, the second painting was evaluated as more creative when it was labeled as human-made and followed a painting labeled as AI-made (see Fig. 2). Once more, this suggests directly comparing art labeled as human and AI-made can increase perceptions of human creativity. That is, the exact same painting was judged to be more creative, novel, likable, and appropriate to be sold in a gallery when it was labeled human-made and compared against an AI-made painting than when it was labeled as human-made and compared against another human-made painting. Note, these results did not change substantively in supplementary regression models used to control for participants’ experience in either artistic or technological industries; models testing for interactions suggested the boost in perceptions of creativity was slightly more pronounced in participants with more artistic experience though the interaction term failed to reach significance (*b* = 0.23, *p* = 0.22). This suggests it is unlikely that the observed effects are primarily driven by anxieties specific to participants whose passions or livelihoods are more directly impacted by generative AI technologies.

**Table 2:** Experiment 4 between-subject evaluation means for Image 2.

	Human-anchor	AI-anchor	Human-anchor
	Human label (control condition)	Human label	AI label
Creative	4.62 (0.08)	4.85 (0.08)*	4.24 (0.08)**
Monetary value	148.35 (11.86)	152.99 (19.73)	111.74 (13.30)***
Estimated time to produce	36.02 (1.43)	32.60 (1.46)	18.29 (1.35)***

Standard errors are reported in parentheses above. *P*-values reflect post-hoc pairwise comparisons using ART-C tests comparing each experimental group to the control condition.

*p < 0.05, **p < 0.01, ***p < 0.001.

## Experiment 5

### Results and discussion

In Experiment 5 (preregistered), we test the generalizability of effects observed in Experiments 3 and 4 by recruiting a representative sample of the US population (*n* = 709) to evaluate a larger set of images. Each participant was asked to evaluate two images randomly selected from those used in our first experiment. Labels were randomized so that participants were assigned to either a control condition (a) where both paintings were labeled as human-made, or an experimental condition (b) where the first painting was labeled as AI-made and the second labeled as human-made. Creativity, monetary value, and production time were all assessed using the same measures from Experiment 4. As a control variable, participants indicated their opinion towards AI using four items (α = 0.77) adapted from the General Attitudes Towards Artificial Intelligence Scale^31^.

**Figure 3:**
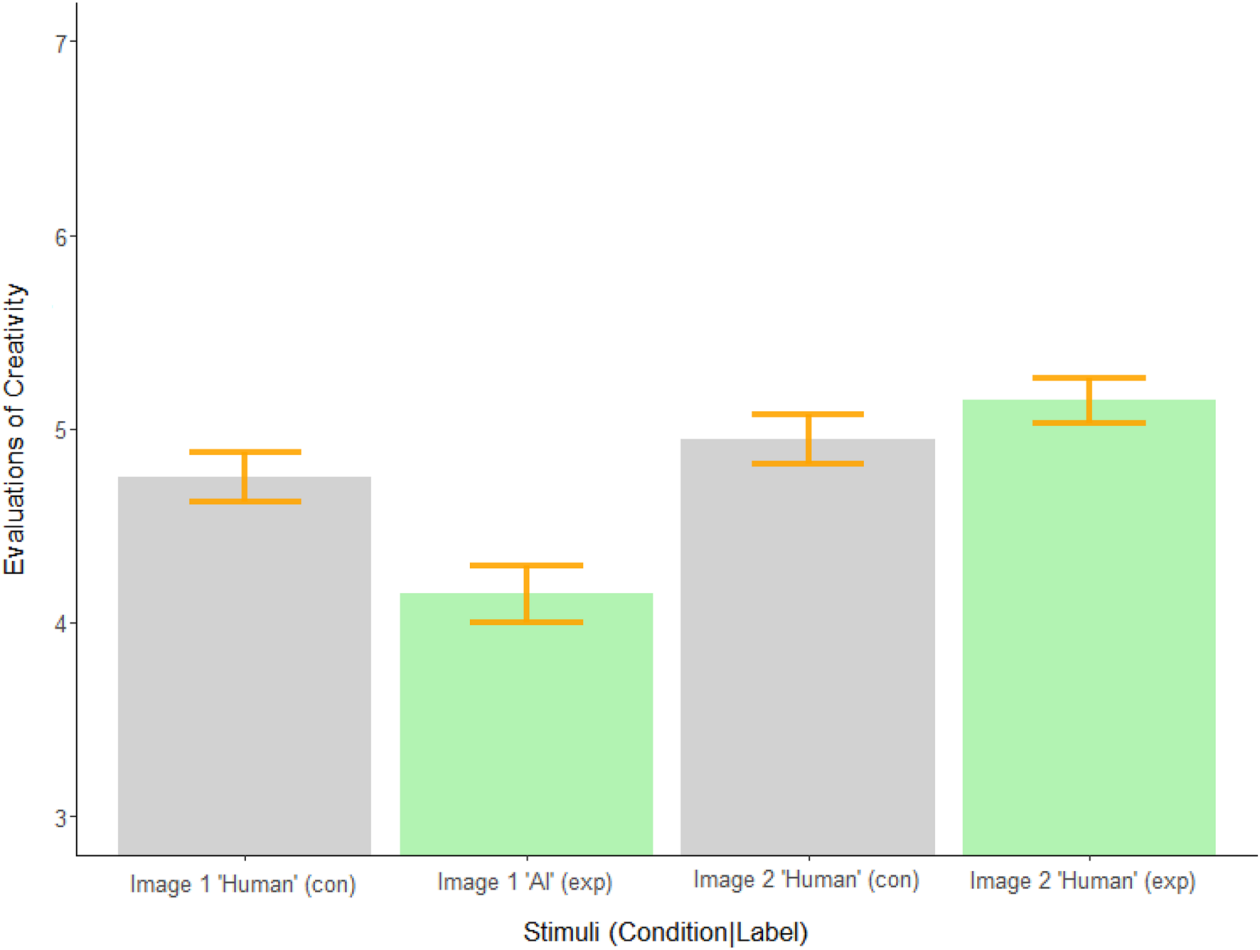
Experiment 5 sequential evaluations of creativity by condition. *Note* Colors correspond to conditions. Grey is used for the control condition which only contained images labeled as human-made. Green is used for the experimental condition which contained an image labeled as AI-made first and an image labeled as human-made second. The error bars represent standard errors. An omnibus ART-C test revealed an overall difference between conditions for evaluations of image 2 (*t*[708] = − 3.084, *p* = 0.002) and post-hoc comparisons found significant differences between evaluations of all images (*p* < 0.01) in the directions shown above with the exception of the two images both labeled as human-made in the control condition (*p* = 0.11). Effects remained unchanged if we used regression models to additionally control for variation attributable to specific images or individuals (e.g., anxiety about AI technologies and artistic experience).

Consistent with the bias against AI-made art documented in earlier studies, the first painting presented to participants was evaluated as less creative, worth less money, and estimated to have taken less time to produce when labeled as AI-made (see Table S6 in our Supplementary Information). Consistent with results observed in Experiment 3, the second painting presented was seen as more creative when labeled as human-made if it followed a painting labeled as AI-made relative to when it followed a painting labeled as human-made (see Table 3 and Fig. 3). The direction and significance of these results was unchanged in supplementary regression models using attitudes towards AI as a control variable; models testing for interactions suggested the boost in perceptions of creativity may be slightly more pronounced for participants with greater anxiety about AI technologies but the interaction term failed to reach significance (*b* = 0.10, *p* = 0.29). This suggests these effects are not primarily driven by a general distaste toward AI. That is, regardless of how participants felt about AI, they generally (a) evaluated AI-made art less favorably and (b) saw human-made art as more creative after evaluating AI-made art.

**Table 3:** Experiment 5 between-subject evaluation means for Image 2.

Dimension	Human anchor	AI anchor	Mean difference
	Image 2 Human Label (Control)	Image 2 Human Label	
Creativity	4.95 (0.06)	5.14 (0.06)	*t*(688.18) = 2.26, *p* = 0.024
Monetary Value	132.87 (6.44)	167.83 (19.33)	*t*(467.25) = 1.72, *p* = 0.087
Estimated Time to Produce	30.51 (1.23)	30.94 (1.18)	*t*(696.81) = 0.25, *p* = 0.81

Means and standard error reported above. *P*-values reflect between-subjects *t*-tests comparing the left and right columns. The direction and significance of these effects remained unchanged if we used OLS regression to additionally control for variation attributable to specific images or individuals (e.g., anxiety about AI technologies and artistic experience). *p < 0.05, **p < 0.01, ***p < 0.001.

## Experiment 6

### Results and discussion

Experiment 6 (preregistered) used a representative sample of the US population (*n* = 527) to test whether the effects observed in our previous experiments extend to art *collaboratively* produced by human artists and AI. To clarify, many works made by AI today require humans to provide specific verbal prompts that direct the AI’s efforts in some way. This might be considered collaborative only in a very strict sense, and was not what we were interested in here. That is, choosing a prompt might be thought of as collaborative in the same way that a human patron might commission another human (with greater artistic skill) to produce a specific artwork without necessarily being granted the shared title of “artist” (e.g., the ceiling of the Sistine Chapel and the Mona Lisa are not typically thought of as collaborations, despite both pieces being commissioned by patrons who dictated some or all of the themes and content therein^32^). Here, however, we were interested in the perception of distributed artistic collaborations—when artists pool their talents and effort to generate a shared product. This distinction is valuable because one can imagine that many AI technologies are currently being adopted to supplement, facilitate, or expedite the creativity of employed human artists who already possess many of the requisite skills needed to produce high quality art on their own. We deemed this exploration important because it is not clear whether collaborations between artists and AI will be subject to the same positive and negative effects observed in our previous experiments*.* That is, will collaborations between human artists and AI artists (which, by their very existence, might be likely to prime cognitive comparisons between humans and AI) be evaluated more or less favorably than art attributed to either party working in isolation?

To examine this question, participants in Experiment 6 rated two randomly ordered paintings (see Fig. S3 in our Supplementary Information) that were newly generated for this experiment and pretested to be comparable in terms of creativity (*p* = 0.77), monetary value (*p* = 0.54), and estimated production time (*p* = 0.60). Participants were randomly assigned to one of two conditions where they were first shown a painting that was either labeled as human-made or AI-made. Subsequently, all participants were shown a second painting that was always labeled as collaboratively made across both conditions. Specifically, participants read the prompt: “The following painting was created by the artist Avery Taylor, collaborating with an artificial intelligence program capable of imagining and painting entirely of its own accord, in January of 2019.” Creativity, monetary value, and estimated production-time were all measured using the same items from Experiment 5. Finally, to test whether humans are seen as more or less *responsible* for creative output when working with an AI, participants estimated the distribution of labor for the collaborative painting using a 100-point slider ranging from “All AI Effort” to “All Human Effort.”

**Figure 4:**
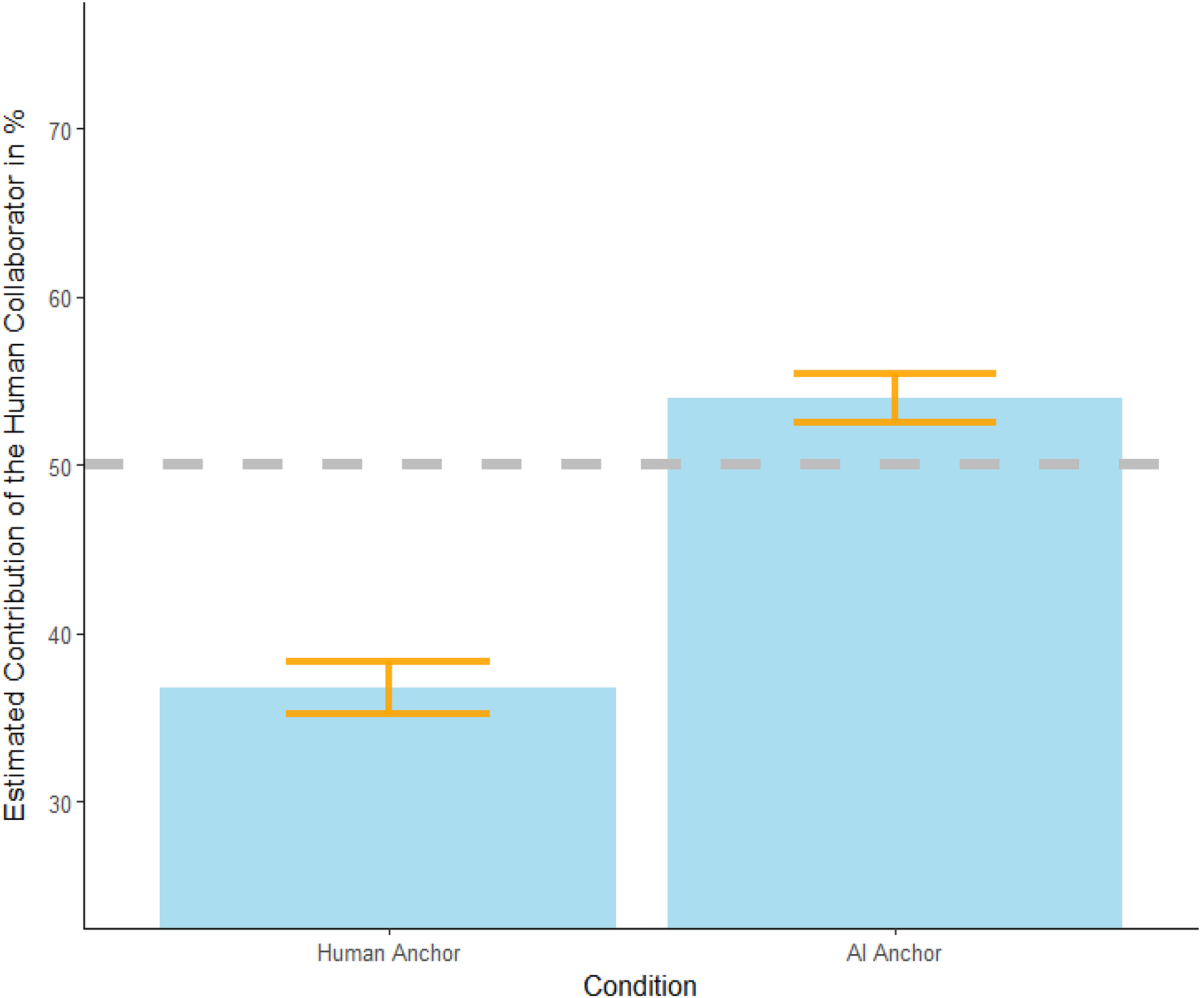
Estimates of workload distribution in a human-AI collaboration. *Note* The y-axis represents the portion of work participants estimated the human was responsible for in their collaboration with an AI technology. The x-axis represents the different conditions (i.e., whether participants evaluated an image labeled as human-made or AI-made first). The gray dotted line is included to illustrate where estimates of an even 50/50 split would land. The error bars represent standard errors.

Consistent with the findings from our previous experiment, the first painting was evaluated less favorably on all dependent variables when labeled as AI-made (see Table S6 in our Supplementary Information). On average, the second painting (always labeled as collaboratively made) was rated less favorably than human-labeled painting and more favorably than AI-labeled painting (see Table 4). Participants also estimated the human artist in this collaboration was responsible for a greater portion of creative labor (53.94%) when the collaboration was compared to a painting labeled as AI-made, but a smaller percentage of creative labor (36.76%) when the collaboration was compared against a painting labeled as human-made: t(514.1) = 9.09, *p* < 0.001, *m*_*diff*_ = 17.18, 95% CI [13.01, 21.35], *d* = 0.71 (see Fig. 4). In sum, these results indicate two important findings. First, the bias against AI-made art persists even when art is a collaborative production of humans and AI working together. Second, estimates regarding human labor in a collaboration with AI depended largely upon if the collaborative piece was being compared to solitary human or AI efforts. That is, when compared against AI-made art, human artists were seen as responsible for more of the creative labor in a collaboration but when compared against human-made art, human artists were seen as responsible for  less of the creative labor in a collaboration.

**Table 4:** Experiment 6 within-subject mean difference between “collaboration” painting and anchor.

Dimension	Collaboration vs. human anchor	Collaboration vs. AI anchor
Creative value	− 0.58 (0.09)***	0.46 (0.07)***
Monetary value	− 126.60 (36.13)***	30.06 (5.25)***
Estimated time to produce	− 16.45 (1.62)***	10.64 (1.03)***

*P*-values and standard error are from paired *t*-tests. Significant effects reported above remained unchanged when additionally including evaluations of image 1 as control variable in an OLS regression model.

*p < 0.05, **p < 0.01, ***p < 0.001.

## General discussion

Do we evaluate art attributed to humans and art attributed to AI similarly? Our findings suggest not. Across six experiments, our results indicate that even when the content of the art is held constant, simply believing the art was made by AI negatively impacts appraisal. This bias against art labeled as AI-made was particularly evident on dimensions of monetary value and skill (see Experiments 1 and 2). On the one hand, these findings are in line with anxieties expressed in popular media outlets that like other industries affected by automation in the past, AI-made art is likely to bring down the average monetary value commanded in artistic markets. Indeed, in some cases, the effects we observe are substantive. For example, AI-made labels in our last experiment led to a 62% decrease in monetary value and a 77% decrease in estimated production-time relative to perceptions of human effort. On the other hand, images labeled as AI-made in our studies were often less liked and less likely to be considered “art”, and our results suggest many of these devaluation effects can be mitigated by ensuring audiences do not directly compare human and AI efforts (see Experiments 2 and 3). Of course, one limitation in many of our experiments is that many of the AI-generated stimuli used were purposefully designed to be indistinguishable from human-made art. That is, we pretested images to ensure any effects we observed would be driven by sources attributions rather than differences in content. In this regard, we can only speculate as to how art that has been produced by less constrained AI will go on to impact the art world at large. Indeed, a small but promising body of work suggests AI art already possess its own style^33^ and has been perceived as *more* creative than contemporary human artists in some cases^34^. However, in light of these findings and our own, it is difficult to imagine how AI will not bring down average price of art in global markets, particularly if markets become saturated with artistic products that are not only cheaper to produce en masse, but less likely to be valued by consumers. Based on these conjectures, new strategies that allow human artists to maintain the market value they currently command may be advisable. For instance, human value may be less subject to change if greater efforts are made to partition art markets, segregating human and AI-made goods more definitively. One simple proposition is to adopt the designation “synthography” to create greater psychological distance between specific forms of human art (like photography or digital art) and comparable works by made by AI^35^.

Will evaluations of human creativity be impacted by comparisons to AI? Our findings suggest yes, but perhaps not always in straightforward ways. For example, we find that although participants consistently devalued art if they believed it was made by AI, art labeled as human-made was seen as more *creative* when it was compared against art labeled as AI-made than when it was compared against art attributed to other humans. This refutes the sensationalized sentiment that “art is dead”^5^ because of the introduction of AI. If anything, our results predict AI art has the potential to invigorate audiences to see human creativity in a new light if used carefully.

How will collaborations between humans and AI (e.g., when human artists use AI tools) be received in creative industries? Here, our findings are nuanced but illustrative. Participants found images labeled as collaborations between a human artist and AI to be more valuable than art labeled as solely AI-made but less valuable than art labeled as solely human-made. Importantly, though, we found that the human’s status as the primary creative agent (i.e., the target more responsible for the output) in a collaboration depended upon whether the collaboration was compared to solitary human or AI efforts. This finding has significant practical value, because maintaining one’s status as the primary creative agent implies the ability to capture a greater portion of economic value. In short, it suggests human artists who use AI-tools might be smart to encourage comparisons between their own art and art made by AI working without the assistance of human artists.

We are only just beginning to understand the impact AI technologies will have on the value of human creativity, but it is worth noting that many heated debates the birth of generative AI has provoked are strikingly reminiscent of initial reactions to the invention of the camera. For example, the famous French painter Paul Delaroche declared in response to the camera, “From today, painting is dead”^36^. Similarly, the famous art critic Charles Baudelaire once said, “it will not be long before it [photography] has supplanted or corrupted art altogether”^37^. In some ways, their anxieties were justified. The livelihoods of nineteenth century portraiture artists were threatened by the invention of the camera^38^. And yet, the camera also gave us new ways to look at the world, defining a period of innovation that eventually led to the creation and appreciation of new forms of art that included impressionism, cubism, and digital photography^39^. Likewise, respected media outlets and artists alike have recently flooded the internet with responses to AI like, “More than ever before…I’m concerned for the future of human creativity”^6,40,41^. Our findings allow us to imagine a different future than these prevailing forecasts of doom and gloom, one where the value of human creativity persists. Just as the invention of photography ultimately inspired innovations like impressionism^42^, we suggest that a heightened appreciation for human creativity can provide a fertile bed for new forms of human creativity and expression.

## Methods

We report all conditions, measures, data exclusions, and provide copies of all study materials on our Open Science Framework page (see Data and Code Availability**)**. Research protocols were approved by the Human Research Protection Office and Institutional Review Board of Columbia University. We confirm that all research was performed in accordance with relevant guidelines and regulations including the Declaration of Helsinki. Participants gave informed consent to participate in all studies. Note, our first two experiments were run in 2017 and 2018, before the more recent introduction of AI-art innovations like Midjourney and DALL-E2. In contrast, our last experiment was run in 2023 just as Midjourney Version 4 and DALL-E2 were beginning to reach a national audience. We share these dates because we believe it is important to note, historically, that our data was collected both before and after more recent (and sensationalized) coverage of AI art in prestigious media outlets like the New York Times^5^ and Washington Post ^6^. That is, our research documents evaluations of art just as the implications of AI artists are beginning to be realized.

### Sample size determination and randomization

All sample sizes were determined before collecting data, with data collection halting after analysis began. For Experiment 1, we hypothesized a small to medium effect size (Cohen’s *d* = 0.33), which we used to determine our sample size (i.e., roughly 95% power to detect an effect). We then used effect sizes from earlier experiments to make sample size determinations for later experiments. Data quality was ensured in several ways. For instance, we removed duplicate responses (e.g., repeated IP addresses or research IDs), participants who failed attention checks, and collected entirely new samples for every study.

### Data analysis and reporting

All data analysis was conducted in R (v.4.2.2). Effect sizes were calculated as Cohen's *d* using the ‘effectsize’ package^43^. Whenever a t test in our analysis did not demonstrate equal variance, a Welch's t-test with corrected degrees of freedom was used instead. Reported *p*-values are all two-sided. Finally, all regression models include dummy-coded conditions that compare outcomes from against the control condition designated for that experimental design.

### Experimental samples and procedures

#### Pilot study

We pre-tested the 28 images used in Experiments 1, 2, and 5 in a pilot study (*n* = 105). This was done to ensure our stimuli captured a range of styles and quality. Half of these images were lesser-known paintings from respected artists (e.g., William Gear, Andy Warhol, and Paul Gauguin) while the other half were AI-generated images rendered in the same styles of those artists. These stimuli were chosen and tested to ensure that (a) participants could not tell the difference between human and AI-made art and (b) so that participants in Experiments 1 and 2 would be presented with style-matched pairs randomly labeled as “human-made” or “AI-made”. Results confirmed images represented a range of quality (*m* = 4.30, *sd* = 1.21) and that participants generally could not tell the difference between images that were or were not AI-made. For example, after evaluating each image on aesthetic dimensions, participants were then asked to guess the origin of each image (1 = *definitely human-made*, 6 = *definitely AI-made*). Responses were skewed (see Fig. S1 in our Supplementary Information), indicating that regardless of each image’s actual origin, participants thought the majority of images were human-made (e.g., the average guesses for images that were, in reality, human-made were comparable to the guesses for images that were, in reality, made using AI programs, *m* = 2.60, *sd* = 1.63 vs. *m* = 2.65, *sd* = 1.64, *p* = 0.59). To ensure our data accurately represented lay evaluations of stimuli participants were unfamiliar with (i.e., experimental fidelity) we used the question “Before taking this survey, had you seen any of these paintings before?” Participants who responded yes to this question in any experiment were removed before any analysis, though supplementary analysis including their responses did not change the direction or significance of effects reported in this article.

#### Experiment 1

We recruited 143 English speaking US residents from Mturk. Participants were excluded for failing to pass attention checks or reporting they recognized stimuli used in the study, yielding a final sample of *n* = 119 participants (men = 52%, $$m_{age} = 34$$). Participants were paid $2 to complete the survey.

After rating three buffer images to acclimate participants to the task, all participants rated 14 images labeled as human-made and 14 images labeled as AI-made. Images were all presented in a random order. To make sure that differences in artistic style did not confound any results, labels were randomly assigned within style-matched pairs (see our Pilot study) such that one image in each style-matched pair was always labeled as AI-made and the other as human-made. This allowed us to make comparisons between images labeled as human or AI-made while holding style constant. Participants then rated each painting on a battery of dimensions: how much they liked it, how skillfully it was painted, how colorful it was, whether they found it inspiring, how bright it was, how complex it was, how emotionally evocative, and whether they thought it was expensive (1 = *Not at all*, 7 = *A great deal*; *α* = 0.88). For exploratory purposes we also asked participants about their general affinity for art (e.g., “Some people seem to need art in their lives more than others; I consider myself that kind of person.”) and their feelings about technological innovations (e.g., “I tend to dislike new technologies.”). Supplementary analysis revealed these had no impact on our main findings.

#### Experiment 2

We recruited 555 English speaking US residents from Mturk. Participants were excluded for failing to pass attention checks, comprehension checks, or reporting they recognized any stimuli used in the experiment, yielding a final sample of *n* = 415 participants (men = 51%, $$m_{age} = 36$$). Participants were paid $2 to complete the survey.

After rating three buffer images to acclimate participants to the task, all participants rated 28 images in random order. Participants were randomly assigned to one of three conditions. In a control condition we made no mention of AI, nor did we label images as “human-made”. That is, we believed it important to have a control condition in one study that did not prime any implicit comparisons of humans and non-humans that might impact evaluations. Instead, images were unlabeled on the presumption that participants would assume the images to be human-made. This presumption was based on our pre-test, where participants indicated the majority of images looked human-made even after being informed them that some were made by AI (Fig. S1 on p.1 of our Supplemental Information). Thus, participants were simply told that we were interested in “how people perceive each painting on a number of dimensions.” In experimental conditions participants were either told “each painting was made by an artificial intelligence” or that “some of these paintings were made by a human and others were made by an artificial intelligence” but not which ones. Participants then rated each painting on a battery of dimensions: how much they liked it, how skillfully it was painted, how colorful it was, whether they found it inspiring, how bright it was, how complex it was, how emotionally evocative, whether they thought it was expensive, and how much they’d be willing to pay (1 = *Not at all*, 7 = *A great deal*; *α* = 0.94). For exploratory purposes we also asked participants about their mood during the study (e.g., “Overall, my mood is:” = − 10 = *Very unpleasant*, 10 = *Very pleasant*) and about their personal tastes in art (e.g., “I feel I have good taste in art.”). Supplementary analysis revealed that these did not differ by condition and had no impact on our main findings.

#### Experiment 3

We recruited 541 English speaking US residents from Mturk. Participants were excluded for failing to pass attention checks, comprehension checks, or reporting they recognized stimuli used in the study, yielding a final sample of *n* = 405 participants (male = 53%, $${m}_{age}=38$$). Participants were paid $1 to complete the survey.

To increase the external validity of our findings, participants were given a cover story that said these images represented real paintings for sale at a private gallery:

*“On the next page, you'll be shown two images of paintings currently for sale at the Lenham Private Gallery. We are curious about consumer impressions of these paintings and the blurbs attached to them. Please review the painting and information provided by the gallery and answer all questions honestly”.*

Participants were then randomly assigned to one of four conditions where they rated two images. In a control condition, both images were labeled as human-made. In one experimental condition both images were labeled as AI-made. In another experimental condition, the first image was labeled human-made and the second AI-made. And in a second experimental condition, the first image was labeled AI-made and the second human-made. Image order and the images themselves were held constant across conditions. Human and AI-made labels read as follows, “The following painting was created by Jamie Kendricks, in January of 2019.” or “The following painting was created by an artificial intelligence program, which imagines and paints images entirely of its own accord, in January of 2019.” Building upon our cover story, both paintings were presented with unique ID numbers and fabricated gallery information (e.g., “Lenham Private Gallery ID: #A2461; Untitled, 2019; Oil on canvas; 24 in × 36 in). Participants rated each image on a battery of dimensions: how much they liked each painting, how skillfully it was painted, how colorful it was, whether they found it inspiring, how bright it was, how complex it was, how emotionally evocative, whether the creator was talented, and whether they were impressed by the execution (1 = *Not at all*, 7 = *A great deal*; $\alpha_{image 1} = 0.86, \,\alpha_{image 1} = 0.90$).

In addition, direct estimates of monetary value were obtained on a separate page immediately after participants evaluated each painting on the dimensions listed above. On this page, participants were informed about pricing with the prompt: “The average painting in the Lenham Gallery sells for somewhere between $50 and $220, with most pieces retailing at $150.” They were then asked, “How much do you personally think the Lenham gallery should sell this painting for?” and “Assuming that you wanted this painting and given the gallery's prices, how much would you pay to acquire it?” For exploratory purposes, we asked participants about their own taste in art (e.g., “Compared to other people, I generally have a better eye for art.” and “I like artwork that depicts "real things" more than I like artwork that is abstract.”). Supplementary analysis revealed artistic taste had no impact on our main findings.

#### Experiment 4

We recruited 792 English speaking US residents from Prolific. Participants were excluded for failing to pass attention checks, comprehension checks, or reporting they recognized any stimuli used in the study, yielding a final sample of *n* = 789 participants (male = 49%, $$m_{age} = 38$$). Participants were paid $1 to complete the survey. Our pre-registration can be found here: https://aspredicted.org/DJV_MN7.

Participants were given the same cover story used in Experiment 3. They were told that we were curious about their impressions of paintings currently for sale at the Lenham Private Gallery and then randomly assigned to one of three conditions where they were asked to evaluate two images. In a control condition, both images were labeled as human-made by using the names of made-up human artists (e.g., “The following painting was created by [Jamie Kendricks or Taylor Jennings], in January of 2019”). In one experimental condition, the first image was labeled human-made and the second AI-made (e.g., “The following painting was created by an artificial intelligence program, which imagines and paints images entirely of its own accord, in January of 2019.”). In another experimental condition, the first image was labeled AI-made and the second human-made. We used the same labels and gallery information provided in Experiment 3. The perceived creativity of each image was measured by asking participants how creative, novel, appropriate (to be sold in a gallery), and likable each image was (1 = *not at all*, 7 = *a great deal*; $\alpha_{image 1} = 0.78,\, \alpha_{image 2} = 0.82$). As in Experiment 3, participants were then given information about pricing on a separate page and asked to estimate the monetary value of each painting. Additionally, participants were asked to estimate labor with the item: “How many hours of active painting time do you think it took to create the painting above?” Finally, to make sure effects were not confounded by individual expertise in domains of art and technology, participants responded to five items about artistic experience (e.g., “I used to [or currently] work in a job that primarily deals with the visual arts [e.g. designer, gallery manager, art dealer].” $\alpha = 0 .79)$ and five items about technological experience (e.g., “I used to [or currently] work in a job that primarily deals with computer programming, data science, or engineering.”  $\alpha = 0.72)$. Expertise did not differ by condition and supplementary analysis revealed it had no impact on our main findings.

#### Experiment 5

We recruited 731 English speaking US residents, using Prolific filters to collect a representative sample of the U.S. population. Participants were excluded for failing to pass attention checks, comprehension checks, or reporting they recognized stimuli used in the study, yielding a final sample of *n* = 710 (male = 48%, $$m_{age} = 45$$). Participants were paid $1 to complete the survey. Our pre-registration can be found here: https://aspredicted.org/GJ4_VS4.

Participants responded to the same survey used in Experiment 4 with two differences. First, images were randomly selected and ordered from the larger pool of 28 pretested images used in Experiments 1 and 2. Second, participants were asked at the end of the survey to indicate their own specific attitudes toward AI using four items ($\alpha = 0 .77)$) adapted from the General Attitudes Towards Artificial Intelligence Scale^31^ (e.g., “I shiver with discomfort when I think about future uses of Artificial Intelligence.” and “I think Artificial Intelligence programs are an exciting new tool for human artists.”; $\alpha = 0.77)$. Notably, though participants who felt anxious about AI technology rated AI-labeled artwork less favorably overall, attitudes towards AI did not differ by condition and supplementary analysis using attitudes towards AI as a control variable had no impact on our main findings.

#### Experiment 6

We recruited 698 English speaking US residents, using Prolific filters to collect a representative sample of the U.S. population. Participants were excluded for failing to pass attention and comprehension checks yielding a final sample of *n* = 527 participants (male = 45%, 49). Participants were paid $1 to complete the survey. Our pre-registration can be found here: https://aspredicted.org/DXP_K4M.

Participants were given the same cover story and prompt used at the beginning of Experiments 4 and 5 (i.e., rating “paintings currently for sale at the Lenham Private Gallery”) before being presented with two images in random order. These images were not drawn from our previous studies but were instead created specifically for this experiment using the AI tool, Midjourney. These stimuli were generated to address a limitation in our previous studies, mainly that the images used were constrained to the styles of specific, and historically well-known artists. That is, we did not allow the AI to really be ‘itself’. Seeking to reduce this concern to a degree, our new stimuli were generated by giving the AI tool Midjourny more ambiguous prompts, asking for both “a creative painting” and “a classical painting”. We chose one image (see our supplementary materials) from the four automatically generated for each prompt at random. We then conducted a pretest on these images to ensure that any experimental effects couldn’t be attributed to intentional content variations that might dominate participant assessments of creativity, monetary value, or estimated production time.

Participants were randomly assigned to one of two conditions. In a control condition, the first image was labeled with the tag: “The following painting was created by Jamie Kendricks, in January of 2019.” In our experimental condition, the first image was labeled with the tag: “The following painting was created by an artificial intelligence program, which imagines and paints images entirely of its own accord, in January of 2019.” For participants in both conditions, the second image was always labeled with the tag: “The following painting was created by the artist Avery Taylor, collaborating with an artificial intelligence program capable of imagining and painting images entirely of its own accord, in January of 2019.” Participants rated both images using the same items from Experiment 5 as well as an additional question, specific to the second image, that asked “how much work do you think was done by the AI vs the human?” using a sliding scale (0 = *All AI Effort*, 100 = *All Human Effort*).

### Supplementary Information

Supplementary Information.

## Data Availability

All data, survey materials, and code are available on the Open Science Framework at https://osf.io/xs8bv/?view_only=96a2b4c29a5b4adf8cca18025e18811c.
