# Supplementary material for: Bias against AI art can enhance perceptions of human creativity
Source: Sci Rep. 2023 Nov 3;13:19001. doi: 10.1038/s41598-023-45202-3 (PMC10624838; doi:10.1038/s41598-023-45202-3)
Supplement: Supplementary file 1 — Supplementary Information. [file 41598_2023_45202_MOESM1_ESM.docx]

**SUPPLEMENTARY INFORMATION**

**Figure S1.**

*Pre-Test of Images Used in Experiment 1, 2, and 5*


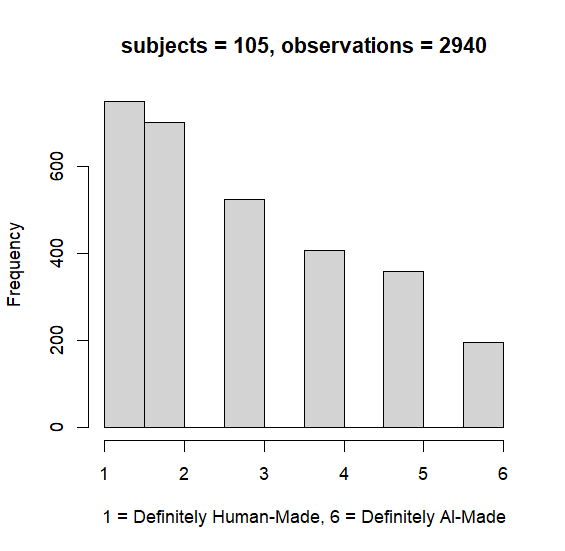


Note. We pretested 28 stimuli used in Experiments 1, 2, and 5. A group of online participants (*n* = 105) were asked to evaluate each image (in random order) on aesthetic dimensions used in Experiment 1. Responses revealed the stimuli represented a range of quality (*m* = 4.30, sd = 1.21). After making those ratings, they were informed that some images they had seen were made by humans and others were made by artificial intelligence but not told which. They were then shown each image a second time (again in random order) and asked to indicate, “How sure are you that a human or artificial intelligence created this painting?” (1 = *Definitely Human*, 6 = *Definitely AI*). As the histogram above shows, when unlabeled, the majority of participants assumed the images were human-made (e.g., 67% of responses were below the midline with an average rating of 2.83 and a median of 3).

**Table S1.**

*Experiment 2 Evaluation Means (Between Subjects)*

| Dimension | Unlabeled  (n = 140) | Labeled AI-Made  (n = 135) | Mystery  (n = 146) |
| --- | --- | --- | --- |
| Expensive | 4.12 (.01) | 3.85 (.02)*** | 4.07 (.02) |
| Skillful | 4.95 (.01) | 4.73 (.02)*** | 4.77 (.01)*** |
| Complex | 4.53 (.01) | 4.53 (.02) | 4.48 (.01) |
| Colorful | 4.26 (.01) | 4.26 (.01) | 4.24 (.02) |
| Liking | 4.03 (.02) | 4.04 (.02) | 4.03 (.02) |
| Emotional | 3.26 (.02) | 3.18 (.02)* | 3.34 (.02)* |
| Bright | 3.99 (.01) | 3.94 (.01) | 3.94 (.01) |
| Inspiring | 3.12 (.02) | 3.18 (.02) | 3.29 (.02)*** |
| Willingness to Pay | 2.97 (.02) | 2.97 (.02) | 2.98 (.02) |
| Qualifies as Art?  (no = 1, maybe = 2, yes = 3) | 2.67 (.01) | 2.56 (.01)*** | 2.66 (.01) |

Note. Standard errors are reported in parentheses above. *P*-values reflect OLS regression models comparing experimental conditions (i.e., “AI-Labels” and “Mystery”) to the control condition that used unlabeled images. These effects remained unchanged when controlling for variation attributable to images (i.e., controlling for image id). **p* < .05, ***p* < .01, ****p* < .001

**Table S2.**

*Experiment 2 Between-Subjects Dunn’s Test Z-Values*

| Dimension | AI Label vs. No Label | AI vs. Mystery | Mystery vs. No Label |
| --- | --- | --- | --- |
| Expensive | -7.06*** | -5.95*** | -1.18 |
| Skillful | -5.59*** | -.52 | -5.16*** |
| Complex | 0.10 | 1.36 | -1.27 |
| Colorful | -0.21 | 0.20 | -0.43 |
| Liking | 0.53 | 0.32 | 0.21 |
| Emotional | -2.35* | -4.53*** | 2.17 |
| Bright | -1.62 | -0.46 | -1.20 |
| Inspiring | 1.21 | -2.99** | 4.25*** |
| Willingness to Pay | -0.17 | 0.41 | -0.59 |
| Qualifies as Art?  (no = 1, maybe = 2, yes = 3) | -7.11*** | -6.70*** | -0.46 |

Note. Bonferroni corrected *p*-values are indicated using asterisks. **p* < .05, ***p* < .01, ****p* < .001

**Figure S2.**

*Experiment 3 Stimuli*


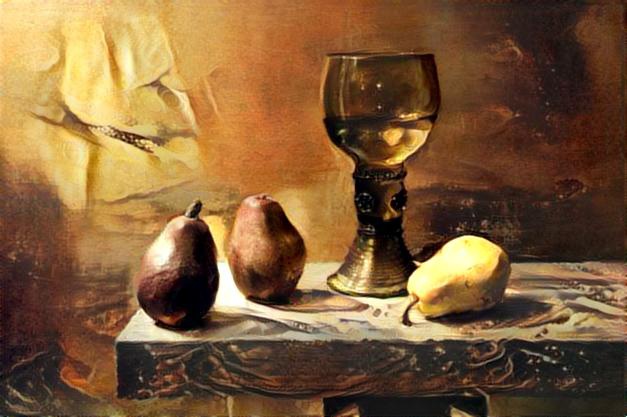

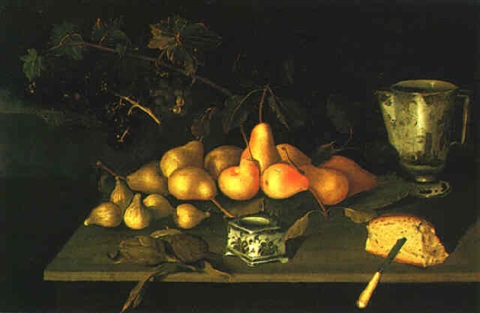


Note. These images were selected from the stimuli used in our previous Experiments because they were rated similarly across artistic dimensions (e.g., evaluations of expensiveness in Experiment 2 were *m* = 4.53, se = .13 and *m* = 4.43, se = .14; *t*[138] = .68, *p* = .50). Please note the image on the left was created using the AI program, DeepDream, which used convolutional neural network to replicate the style of the famed Dutch painter, Pieter Claesz (1597-1660). The image on the right is a painting attributed to the famed Spanish painter Juan van der Hamen (1596 –1631). These images are in the public domain.

**Table S3.**

*Experiment 3 Between-Subjects Evaluation Means for Image 1*

| Dimension | Human-Label | AI-Label |
| --- | --- | --- |
| Aesthetic Dimensions  (*a =* .86) | 4.07 (.05) | 4.01 (.05) |
| Source Value  (*a =* .78) | 5.71 (.05) | 5.09 (.06)*** |
| Monetary Value | $171.38 (9.90) | $110.64 (2.57)*** |

Note. Standard errors are reported in parentheses above. To delineate between aesthetic dimensions on an image (e.g., color and complexity of the image) and attributions about the artist we use the term “source value” above to refer to an aggregation of evaluations of artist skill, talent, and execution. *P*-values reflect post-hoc pairwise comparisons using ART-C tests. **p* < .05, ***p* < .01, ****p* < .001

**Table S4.**

*Experiment 3 Between-Subjects Evaluation Means for Image 2*

|  | Human-Anchor | | AI-Anchor | |
| --- | --- | --- | --- | --- |
| Dimension | Image 2  Human Label  (Control) | Image 2  AI Label | Image 2  Human Label | Image 2  AI Label |
| Aesthetic Dimensions  (*a =* .90) | 3.72 (.11) | 3.69 (.13) | 4.21 (.11)** | 3.78 (.10) |
| Source Value  (*a =* .87) | 5.31 (.11) | 4.52 (.17)*** | 5.56 (.11) | 4.85 (.13)** |
| Monetary Value | $152.87 (20.71) | $97.05 (7.56)*** | $141.63 (5.08) | $109.83 (4.77)** |

Note. Standard errors are reported in parentheses above. Aggregated evaluations of artist skill, talent, and execution are referred to as “source value” above. *P*-values reflect post-hoc pairwise comparisons using ART-C tests comparing each experimental group to the “human-anchor + human-label” control group. **p* < .05, ***p* < .01, ****p* < .001

**Table S5.**

*Experiment 4 Between-Subjects Evaluation Means for Image 1*

| Dimension | Human-Label | AI-Label |
| --- | --- | --- |
| Creativity | 4.60 (.05) | 4.15 (.08)*** |
| Monetary Value | 156.28 (12.03) | 93.56 (5.46)*** |
| Estimated Time to Produce | 32.30 (.90) | 10.04 (1.01)*** |

Note. Standard errors are reported in parentheses above. *P*-values reflect post-hoc pairwise comparisons using ART-C tests. **p* < .05, ***p* < .01, ****p* < .001

**Table S6.**

*Experiment 5 Between-Subjects Evaluation Means for Image 1 (i.e., the Achor Image)*

| Dimension | Human-Label | AI-Label | Mean Difference |
| --- | --- | --- | --- |
| Creativity | 4.75 (.06) | 4.15 (.07) | *t*(706.57) = 6.17, *p* < .0001 |
| Monetary Value in Dollars | 142.21 (17.88) | 80.70 (3.28) | *t*(346.07) = 3.40, *p* = .0008 |
| Estimated Time to Produce in Hours | 23.25 (1.19) | 6.50 (.58) | *t*(471.62) = 17.54, *p* < .0001 |

Note. Standard errors are reported in parentheses above. The direction and significance of these effects remained unchanged when we used OLS regression to additionally control for variation attributable to images and individuals (e.g., anxiety about AI technologies and artistic experience).

**Figure S3.**

*Experiment 6 Stimuli*


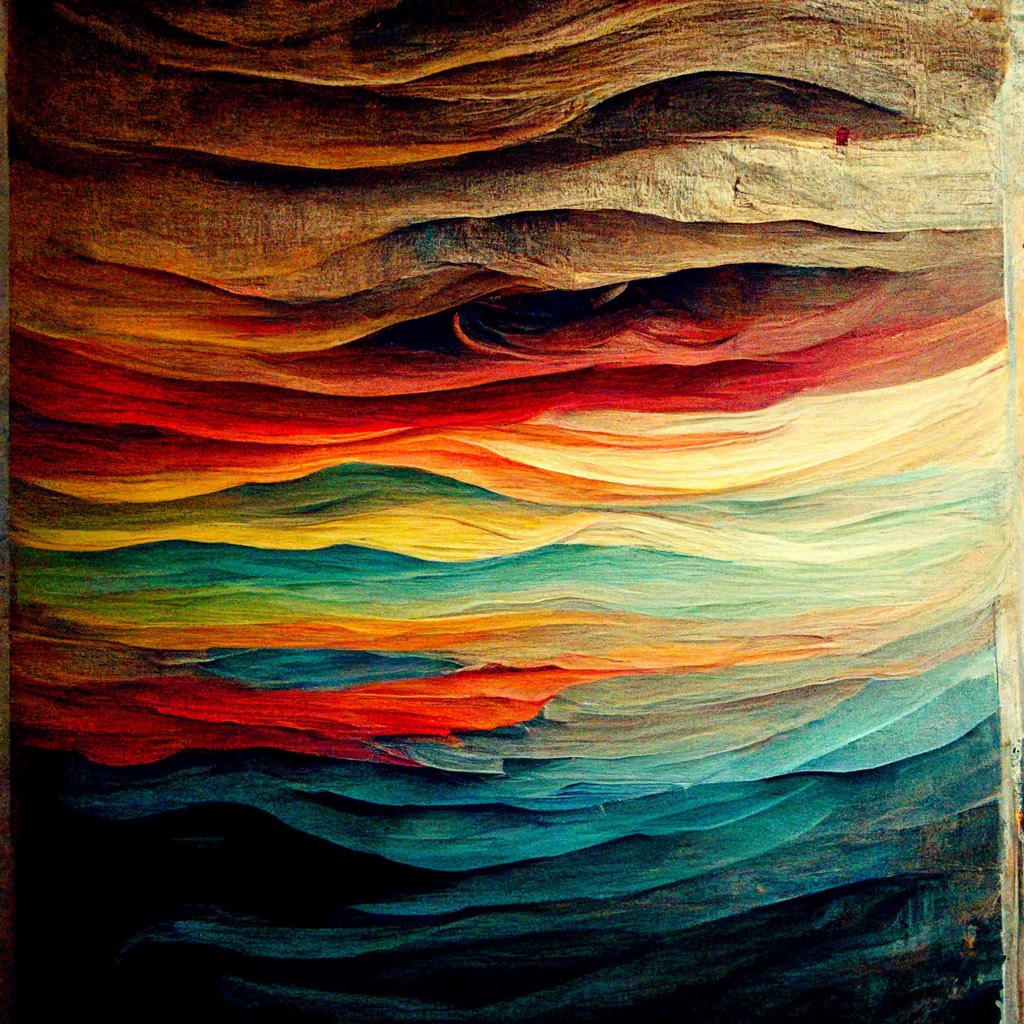

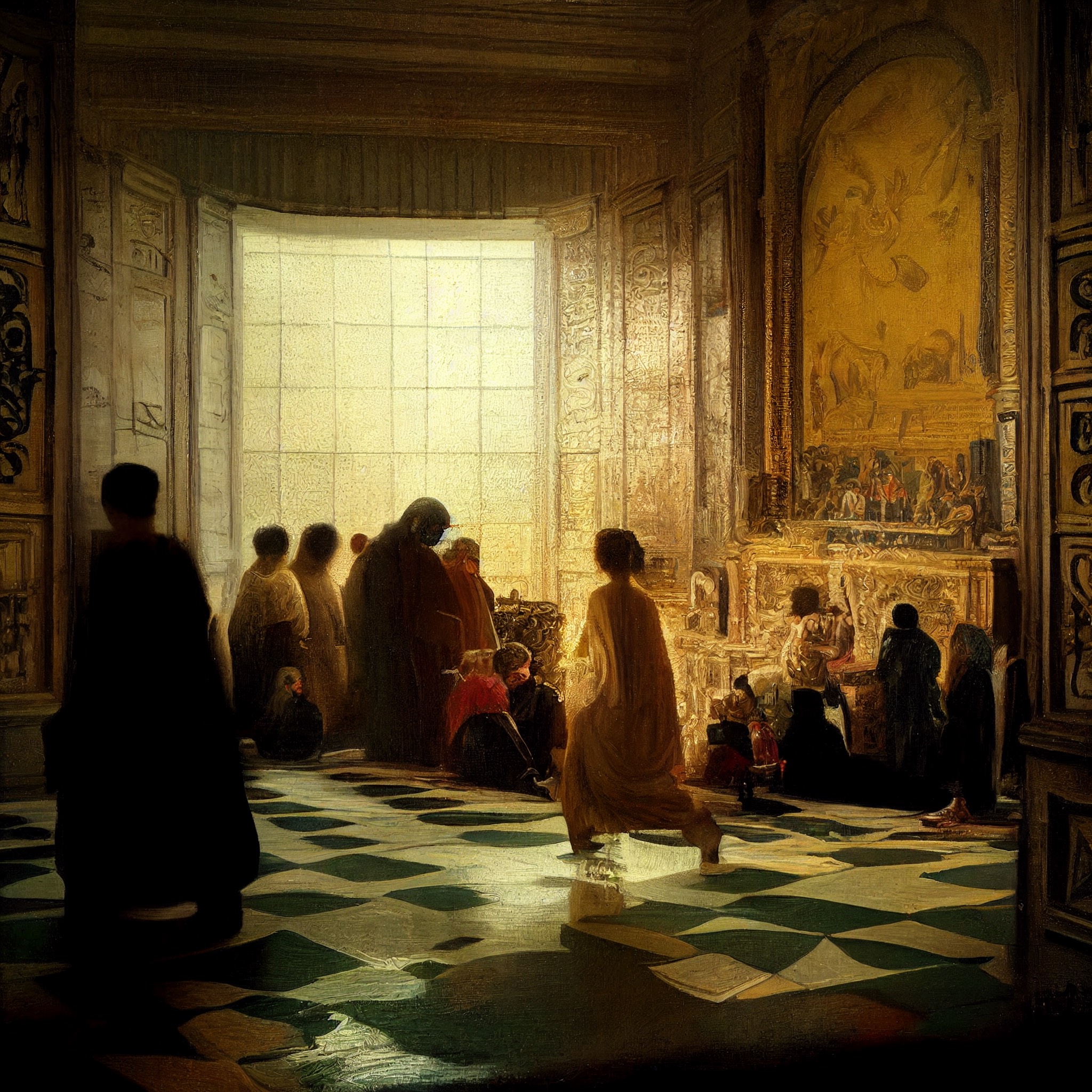


Note. These images were generated using Midjourney V4 by providing the prompts “a creative painting” and “a classical painting”.

**Table S7.**

*Experiment 6 Between-Subjects Evaluation Means for Image 1*

| Dimension | Human-Label | AI-Label | Mean Difference |  |
| --- | --- | --- | --- | --- |
| Creative | 5.44 (.06) | 4.60 (.08) | *t*(515.59) = -8.37, *p* < .0001 |  |
| Monetary Value | 239.28 (37.66) | 90.93 (3.32) | *t*(244.74) = -3.92, *p* = .0001 |  |
| Estimated Time to Produce | 33.55 (1.46) | 7.81 (.80) | *t*(377.28) = -15.46, *p* < .0001 |  |

Note. *P*-values reflect *t* tests. Standard errors reflect OLS regression models estimating means. Effects were unchanged when including self-reported anxiety about AI (which was not impacted by the condition).
